# Optimization Design and Performance Study of Wearable Thermoelectric Device Using Phase Change Material as Heat Sink

**DOI:** 10.3390/ma17133266

**Published:** 2024-07-02

**Authors:** Jiakai Xin, Guiying Xu, Tao Guo, Bohang Nan

**Affiliations:** Beijing Municipal Key Laboratory of Advanced Energy Materials and Technology, School of Materials Science and Engineering, University of Science and Technology Beijing, Beijing 100083, China; 15313135265@163.com (J.X.); gt372328@163.com (T.G.); ustbnbh@163.com (B.N.)

**Keywords:** thermoelectric generator, phase change material, simulation, wearable, output performance

## Abstract

Wearable thermoelectric generators have great potential to provide power for smart electronic wearable devices and miniature sensors by harnessing the temperature difference between the human body and the environment. However, the Thomson effect, the Joule effect, and heat conduction can cause a decrease in the temperature difference across the thermoelectric generator during operation. In this paper, phase change materials (PCMs) were employed as the heat sink for the thermoelectric generator, and the COMSOL software 6.1 was utilized to simulate and optimize the power generation processes within the heat sink. The results indicated that with a PCM height of 40 mm, phase transition temperature of 293 K, latent heat of 200 kJ/kg, phase transition temperature interval of 5 K, thermal conductivity of 50 W/(m·K), isobaric heat capacity of 2000 J/(Kg·K), density of 1000 kg/m^3^, and convective heat transfer coefficient of 10 W/(m·K), the device can maintain a temperature difference of 18–10 K for 1930 s when the thermoelectric leg height is 1.6 mm, and 3760 s when the thermoelectric leg height is 2.7 mm. These results demonstrate the correlation between the device’s output performance and the dimensions and performance parameters of the PCM heat sink, thereby validating the feasibility of employing the PCM heat sink and the necessity for systematic investigations.

## 1. Introduction

Energy serves as an indispensable material foundation for human societal development. Due to the finite reserves of fossil energy on Earth, given the extensive extraction and rapid consumption of fossil energy resources, the issue of energy crisis has become a critical international concern that no country can ignore. To address these challenges, energy innovation, optimized energy utilization structures, and enhanced energy efficiency have become imperative [1,2]. Thermoelectric materials enable the conversion between thermal energy and electrical energy and offer advantages such as environmental friendliness, long lifespan, controllability, small size, robustness, lightweight, noiselessness, and low maintenance requirements [3,4,5,6,7]. With the rapid development of intelligent wearable electronic devices in the 21st century, wearable thermoelectric generators (TEG) have emerged. However, the heat losses in the system during the operation of thermoelectric generators reduce the temperature difference across the generator, thereby lowering the thermoelectric conversion efficiency. These losses occur at the interfaces between the thermoelectric generator and the air, as well as between thermoelectric generators and heat sources.

Phase change materials (PCMs) are substances whose physical properties change with temperature and can provide heat energy. During this process, PCM absorbs or releases a substantial amount of thermal energy [8,9,10]. By utilizing PCM as a heat sink for thermoelectric devices, it is possible to greatly enhance the temperature difference between the two ends of the device, thus improving energy conversion efficiency. In comparison to other materials, PCM offers advantages such as low density, high latent heat, strong thermal storage capacity, and reusability [11].

The feasibility of utilizing PCM as a heat sink for TEGs was initially demonstrated in reference [12]. Lee et al. [12] arranged PCM blocks in an array and achieved excellent flexibility by filling an elastomer between the PCM blocks. Through this approach, the research team measured an output power density of approximately 20 μW·cm^−2^ generated by flexible thermoelectric generators, maintained for 33 min, thus demonstrating the feasibility of utilizing flexible phase change material heat sinks for thermoelectric generators. References [13,14,15] reported research outcomes on using PCM on the hot side of TEGs to improve their thermal stability. Lourdu Jame et al. [13] discovered that placing a PCM heat sink on the hot side of a thermoelectric generator can maintain a stable heat source temperature and offer the feasibility of replacing the heat source after its removal. Maciej Jaworski et al. [14] confirmed the potential application of PCMs as heat sinks for TEGs or as alternatives to TEG heat sources. Tuoi et al. [15] proposed the use of PCM (Polyglycol E600) as a heat sink during the daytime and aluminum fins as a heat source during the daytime. By comparing the output power of TEGs with and without PCM, the study affirmed the feasibility of enhancing energy conversion efficiency through the combination of PCM and TEG.

The literature [16,17,18] reports the simultaneous application of PCMs to both the hot and cold sides of a thermoelectric generator to improve temperature stability on the hot side and enhance heat dissipation on the low-temperature side. S. Ahmadi Atouei et al. [17] found that applying PCM to the hot side of the TEG module not only extended the time for voltage generation after removing external heat sources but also protected the TEG from failures caused by high input heat power. Jing-Hui Meng et al. [18] explored three different placements of PCM on the cold side, the hot side, and both sides of the thermoelectric generator. The results indicated that (1) when PCM was used on the hot side, its ability to store heat effectively protected the TEG from failure due to high temperature; (2) the design of metal fins inside PCM significantly enhanced the temperature difference between the cold and hot sides of the TEG, resulting in a notable improvement in system output performance; (3) compared to the hot side or both sides design, placing the PCM on the cold side of the TEG yielded the best system output performance but the lowest interference resistance. Taking all factors into consideration, the dual-sided design was recommended in practice; (4) increasing the convective heat transfer coefficient at the cold end of the TEG significantly improved the performance of the PCM-TEG system. Saeed Ahmadi Atouei et al. [16] installed the TEG module between a PCM heat sink, which served as the cooling system, and an electric heater acting as the heat source, termed a single-stage structure. Since PCM can store a certain amount of heat, the PCM was used as the heat source for the second-stage TEG. In the second stage, five smaller TEGs were mounted outside the PCM, with PCM acting as the high-temperature side and the alumina fins acting as the heat sink at the low-temperature side, termed a two-stage structure. The results demonstrated that the proposed two-stage thermoelectric generator system produced, on average, 27% higher open-circuit voltage compared to the single-stage TEG system.

References [19,20,21] discussed the utilization of PCM in combination with porous media and paraffin/expanded graphite (EG) composites, which have high thermal conductivity. Thermal conductivity is a key characteristic of PCM that affects its heat transfer capacity and responsiveness. The literature explored various techniques to enhance PCM performance, including nanoparticle additions, expanded graphite additions, preparation of eutectic PCM, and porous metal foams, to improve its thermal conductivity and subsequently enhance the performance of TEG heat sinks. Reference [22] investigated the impact of PCM quantity on improving TEG heat dissipation and demonstrated that a larger quantity leads to greater heat storage and an increased temperature difference between the two ends of the TEG, thereby improving energy conversion efficiency. Reference [23] investigated the influence of the shape of the PCM enclosure on the performance of PCM heat sinks. Zhang Yuqi et al. [23] conducted a study involving the design of four different enclosure structures: honeycomb cavities, fin cavities, hollow cavities, and heat pipe cavities, all filled with PCM with a melting point of 52 °C as a heat sink. Thus, the honeycomb shape was identified as the optimal configuration for the heat sink. Reference [24] investigated the impact of TE (thermoelectric) leg dimensions on device output performance. Liu Youhong et al. [24] employed simulation methods to investigate the influence of the thermal conductivity of encapsulation materials and the geometric parameters of the TE leg on the performance of flexible wearable thermoelectric generators. Subsequently, by optimizing the TE leg height, a 598.1% improvement in maximum output power (169.97 μW) was achieved at a temperature difference of 15 K compared to the original design. In studies on wearable thermoelectric generators, it is ideal for the phase transition temperature of the PCM to be around room temperature or body temperature, with an optimal range of 18–30 °C. These research findings demonstrate that utilizing PCM as the cold end heat sink significantly increases the temperature difference across the TEG, thereby enhancing energy conversion efficiency. Compared to other materials, phase change materials have several advantages, including low density, high latent heat of phase transition, and strong heat storage capacity.

In conclusion, current research has focused on applying PCM heat sinks to both the cold and hot ends of thermoelectric generator devices, providing initial validation for the feasibility of using phase change materials as thermal stabilizers for the high-temperature end and heat sinks for the low-temperature end in thermoelectric generators. The impact of the enclosure shape on the heat sink performance has been studied, and the influence of the quantity of PCM and thermal conductivity on heat sink performance has been analyzed. However, practical applications of PCM heat sinks necessitate consideration of factors such as phase transition temperature, small supercooling or overheating phenomenon in the phase transition process, high latent heat, sufficient latent heat per unit mass or volume, and stable chemical and physical properties. Consequently, comprehensive research on the systematic quantification of PCM heat sinks in thermoelectric generators is urgently needed, as it lacks an optimized design and theoretical foundation to enhance thermoelectric conversion efficiency. Therefore, this paper aims to provide a systematic quantitative analysis of thermoelectric generator PCM heat sinks. By employing PCM as the cold-end heat sink, and by utilizing a bismuth-telluride-based alloy, which exhibits the best performance at room temperature, as the thermoelectric leg material for wearable thermoelectric generators, COMSOL Multiphysics 6.1 software is employed for simulation and calculation. The study analyzes various parameters of the PCM including quantity, phase transition temperature, latent heat, phase transition temperature interval, thermal conductivity, isobaric specific heat capacity, density, convective heat transfer coefficient, and the parameter of the thermoelectric leg, while investigating their underlying mechanisms of action. This research will provide a basis for optimal design and parameter selection in the development of PCM heat sink systems for wearable thermoelectric generators.

## 2. Structural Description and Material Properties

### 2.1. TED (Thermoelectric Devices) Structure of a Compact PCM Heat Sink

**Figure 1:**
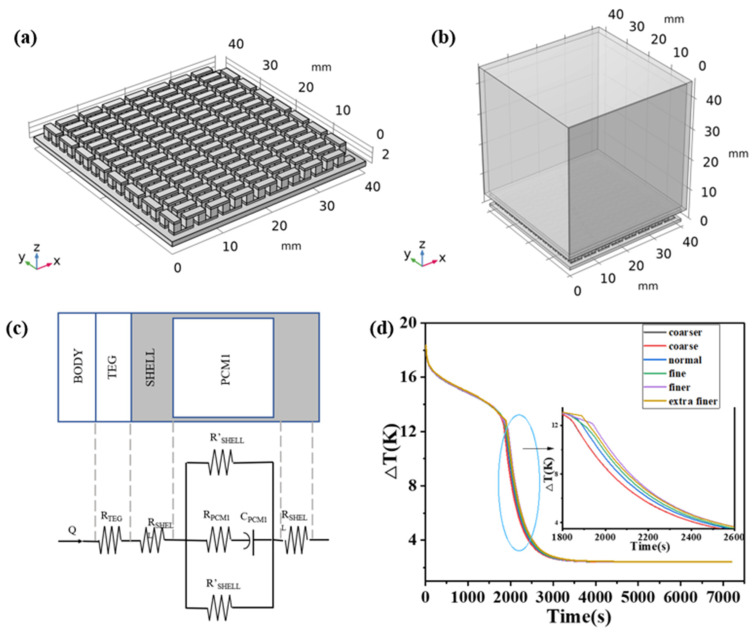
(**a**) The overall structure of the thermoelectric device. (**b**) The internal structure of the thermoelectric generator. (**c**) Geometric partial equivalent thermal resistance diagram. (**d**) Comparison of temperature differences under different grids.

Figure 1a,b presents schematic illustrations of the thermoelectric device structure and the internal structure of the thermoelectric generator chip, respectively. The thermoelectric legs and flow deflectors are located between two alumina plates, with 127 pairs of thermoelectric π elements. The geometric parameters of the thermoelectric legs are listed in the table, and each flow deflector connects a neighboring pair of thermoelectric legs. The PCM is stored in a cuboidal copper enclosure, seamlessly or with zero thermal resistance junction to the thermoelectric generator chip, forming the thermoelectric device. The final dimensions and structure of the geometric modeling are depicted in Figure 1a. The size of the material are shown in Table 1.

### 2.2. Properties of Materials

**Figure 2:**
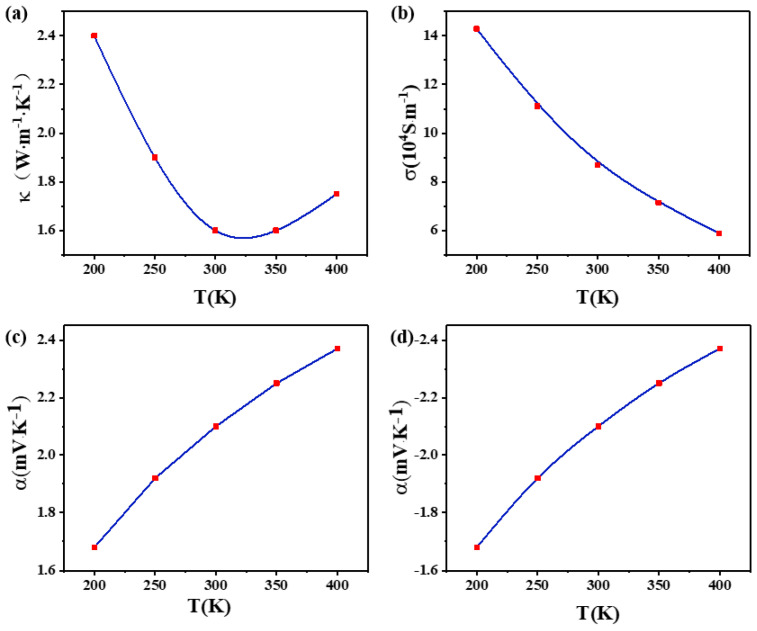
Temperature dependence of (**a**) k(T), (**b**) σ (T), (**c**) α(T), and (**d**) −α(T).

The performance parameters of the material used in the thermoelectric devices are shown in Table 2, among which Seebeck coefficient, electrical conductivity and thermal conductivity are shown in Figure 2.

## 3. Methods

In the simulation, we aimed to investigate the effects of varying parameters such as phase transition temperature, latent heat of phase transition, phase transition temperature interval, thermal conductivity, isobaric heat capacity, density, and convective heat transfer coefficient on the performance of PCM heat sinks and thermoelectric devices. The ultimate objective was to identify an optimal parameter model.

### 3.1. Heat Transfer between Solids and Fluids

The research governing equation is as follows:

(1) $$\rho C_{p}\frac{\partial T}{\partial t}+\rho C_{p}u\cdot \nabla T+\nabla \cdot q=Q+Q_{ted}$$

(2) q=−k∇T

where ρ is the solid density, $C_{p}$ is the constant pressure heat capacity, T is the temperature, t is the time, k is the thermal conductivity, Q is the heat source, $Q_{ted}$ is the thermoelastic damping, an energy dissipation mode, u is the velocity field, q is the heat flux, and ∇ is the gradient operator.

The heat flux equation is

(3) $$-n\cdot q=q_{0}$$

(4) $$q_{0}=h(T_{ext}-T)$$

where h is the convective heat transfer coefficient, $T_{ext}$ is the external temperature, n is the unit vector, and $q_{0}$ is the heat flux in a certain direction.

The boundary heat source equation is

(5) $$-n\cdot q=Q_{b}$$

(6) $$Q_{b}=\frac{P_{b}}{A}$$

where $P_{b}$ is the heat consumption rate, $Q_{b}$ is the heat flux in a certain direction, and A is the boundary area.

The equation of phase transition is

(7) $$\rho =\rho_{solid}$$

(8) $$C_{p}=\theta_{1}C_{p,1}+\theta_{2}C_{p,2}+L_{1\to 2}\frac{\partial \alpha_{m}}{\partial T}$$

(9) $$\alpha_{m}=\frac{1}{2}\frac{\theta_{2}-\theta_{1}}{\theta_{1}+\theta_{2}}$$

(10) $$k=\theta_{1}k_{1}+\theta_{1}k_{2}$$

(11) $$\theta_{1}{+\theta }_{2}=1$$

where $\rho_{solid}$ is the density of the solid material, $L_{1\to 2}$ is the latent heat released per unit mass during the phase transition process, $\theta_{1}$ is the percentage content of phase 1, $\theta_{2}$ is the percentage content of phase 2, $k_{1}$ is the thermal conductivity of phase 1, $k_{2}$ is the thermal conductivity of phase 2, and $C_{p,1}$ is the constant pressure heat capacity of phase 1. $C_{p,2}$ is the constant pressure heat capacity of phase 2.

### 3.2. Thermal Resistance Model and Formula

In order to explain the mechanism and formula of the heat transfer process of the structure, this paper proposes an equivalent thermal resistance model similar to the circuit and gives the corresponding heat transfer equation. PCMs will absorb a lot of heat when phase change occurs. Similar to capacitors in circuits, PCMs can be calculated as heat containers:

(12) $$R=\frac{H}{W\cdot L\cdot k}$$

(13) $$C=H\cdot W\cdot L\cdot \rho \cdot C_{PCM}$$

where *R* and *C* are thermal resistance and thermal capacity respectively, $C_{PCM}$ is the specific heat capacity of the phase change material at different temperatures, *H* is the height of the material in the heat flow direction, *W* is the width of the material in the heat flow direction and Lis the length of the material in the heat flow direction.

(14) $$C_{PCM}=\left\{\begin{array}{c} C_{p},\;T\leq T_{melt}\\ C_{p}+\frac{L}{∆T},T_{melt}\leq T\leq T_{melt}+∆T\\ C_{p},\;T\geq T_{melt}+∆T \end{array}\right.$$

The energy balance equation for TEG is as follows:

(15) $$Q_{h}=\alpha T_{h}I_{TEG}+k_{TEG}\left(T_{h}-T_{c}\right)-\frac{1}{2}R_{e}I_{TEG}^{2}$$

(16) $$Q_{c}=\alpha T_{c}I_{TEG}+k_{TEG}\left(T_{h}-T_{c}\right)+\frac{1}{2}R_{e}I_{TEG}^{2}$$

where $Q_{h}$ is the heat absorbed by the hot end of TEG, and $Q_{c}$ is the heat discharged by the cold end of TEG.

Power generation efficiency can be expressed as

(17) $$\eta =\frac{I_{TEG}^{2}R_{L}}{\alpha T_{h}I_{TEG}+k_{TEG}\left(T_{h}-T_{c}\right)-\frac{1}{2}R_{e}I_{TEG}^{2}}$$

When the load is connected, the external voltage of the generator is

(18) $$V=I_{TEG}R_{L}=\alpha \left(T_{h}-T_{c}\right)-I_{TEG}R_{e}$$

The output power of the thermoelectric generator is

(19) $$P=Q_{h}-Q_{c}=\alpha \left(T_{h}-T_{c}\right)I_{TEG}-R_{e}I_{TEG}^{2}=I_{TEG}^{2}R_{L}=VI_{TEG}$$

(20) $$P={\left[\frac{\alpha \left(T_{h}-T_{c}\right)}{R_{e}+R_{L}}\right]}^{2}R_{L}$$

When the internal and external resistors are the same, the maximum output power value is

(21) $$P_{max}=\frac{\alpha^{2}{\left(T_{h}-T_{c}\right)}^{2}}{4R_{e}}$$

For composite phase change materials with graphene added,

(22) $$L_{c}=L_{pcm}(1-w)$$

where $L_{c}$ is the latent heat of the composite phase change material, $L_{pcm}$ is the latent heat of the original phase change material, and w is the mass fraction of the added graphene.

(23) $$k_{c}=V_{g}k_{g}+(1-m)k_{PCM}$$

where $k_{c}$ is the thermal conductivity of composite phase change materials, $V_{g}$ is the volume fraction of graphene, $k_{g}$ is the thermal conductivity of graphene, and $k_{PCM}$ is the thermal conductivity of phase change materials.

### 3.3. Electric Current

(24) $$\nabla \cdot J=Q_{j,v}$$

(25) J=σ·E+σα∇T

(26) E=−∇V

where *E* is the electric field intensity, *V* is the potential, *D* is the electric displacement, *J* is the current density, $Q_{j,v}$ is the rate of change of charge density at points in space with respect to time, t is time, σ is Electric conductivity, and α is the Seebeck coefficient.

(27) $$∆V=\alpha_{ab}(T_{h}-T_{c})$$

where ∆V is Seebeck voltage, $\alpha_{ab}$ is the Seebeck coefficient difference between the two materials, $T_{h}$ is the temperature at the high-temperature end, and $T_{c}$ is the temperature at the low-temperature end.

### 3.4. Thermal and Electrical Coupling Modules

The thermoelectric constitutive equation coupling is as follows:

(28) $$\vec{q}=T\left[s\right]\vec{j}-\left[k\right]\nabla T$$

where $\vec{q}$ is the heat flux vector, $\left[s\right]$ is the Seebeck coefficient matrix, $\vec{J}$ is the current density vector, $\left[k\right]$ is the thermal conductivity matrix, and $\left[\sigma \right]$ is the electrical conductivity matrix.

(29) $$\vec{J}=\left[\sigma \right](\vec{E}-\left[s\right]\nabla T)$$

(30) $$\vec{E}=-\nabla \varphi$$

where $\vec{E}$ is the electric field strength vector and φ is the electric scalar potential.

(31) $$\rho C_{P}\frac{\partial \varphi }{\partial t}+\nabla (T\left[s\right]\vec{J})-\nabla (\left[s\right]\nabla T)=\dot{q}$$

where $\dot{q}$ is the heat generation rate per unit volume.

(32) $$\nabla \left(\left[\varepsilon \right]\nabla \frac{\partial \varphi }{\partial t}\right)+\nabla \left(\left[s\right]\left[\sigma \right]\nabla T\right)+\nabla \left(\left[\sigma \right]\left[\varphi \right]\right)=0$$

where $\left[\varepsilon \right]$ is the dielectric constant matrix.

### 3.5. Boundary Condition

The overall temperature of the thermoelectric element is set to exhibit a decreasing temperature gradient from top to bottom, starting from 309 K and decreasing to 289 K. The initial temperatures for other solid materials are set at 293.15 K, while the initial temperature for the PCM is set at 289.15 K. The ideal model is that the heat flows from the high-temperature end of the thermoelectric leg to the low-temperature end as efficiently as possible. Therefore, the side part of the thermoelectric generator is set to be thermal insulation. while the convective heat transfer coefficient is specified as 10 W/(m^2^·K).

In order to accurately simulate the working state of the device, the following boundary conditions are adopted, and some assumptions are considered to simplify the model so that the deviation between the model and the actual situation is minimized.

(a) Steady-state operation is assumed;
(b) Thin encapsulation materials surrounding the thermoelectric generator are neglected;
(c) Heat flux exists only at the boundary of the encapsulation materials, with heat conduction occurring only along the height direction of the thermoelectric leg. The lateral surfaces of the thermoelectric leg are insulated;
(d) Extremely weak thermal radiation and thermal resistance between different materials are ignored;
(e) It is assumed that all materials exhibit isotropic and constant thermal characteristics.

The chosen physical fields for analysis include both the thermoelectric effect and electromagnetic heat with coupling between the electrical current interface and the solid heat transfer interface.

### 3.6. Model Verification

The geometric model of the materials is divided into grids. In finite element analysis, the divided grid serves as the smallest computational unit, where equations are solved by the computer in each unit. A finer and denser grid leads to more accurate results but also increases computational complexity and time.

In order to exclude the effect of meshing on the results, the effect of meshing on the results was verified. The meshing of the two models of the height of the thermoelectric leg can choose the same grid accuracy. This paper employs six different grids (coarsened, slightly coarsened, regular, refined, slightly refined, and highly refined) for thermoelectric legs with a height of 1.6 mm to investigate grid independence. The results are shown in Table 3.

From Figure 1d, it can be seen that different mesh divisions have varying effects on the computed results. During the time period from the 1800 s to 2000 s, the coarse and coarser meshes produce similar and relatively small temperature differences. As the mesh is refined, the temperature difference maintained by the PCM during this time period increases. However, the finer and extra fine meshes deviate from this trend; under the division of the finer mesh, the temperature difference maintained by the device during the 1800 s to 2000 s period is larger than that of the extra fine mesh. By the 7200 s, the PCM has completely finished the phase transition, and the operating temperature difference of the device remains constant. However, the open circuit voltage increases with mesh refinement, while the temperature difference gradually decreases. This is because the temperature difference is between the two ends of the thermoelectric leg. For different meshing, the temperature calculation results at both ends of the thermoelectric leg are also different, and the relationship between the Seebeck coefficient and the temperature may also be part of the reason. In conclusion, under different grid divisions, the difference between the calculation results is very small, and the change of open circuit voltage is inversely proportional to the change in temperature difference, which is a normal phenomenon. For the calculation results of the normal grid, its average element quality is quite different from that of the coarser and coarser grids, and the average element quality of the finer grid division is little improved for the normal grid. Considering the calculation time and accuracy, the model is divided into normal grids.

## 4. Results and Discussion

We set a fixed area of 40 × 40 mm^2^ for the TEG and heat sink, a height of 40 mm for the PCM, a phase transition temperature of 293 K, a latent heat of phase transition of 200 kJ/kg, a phase transition temperature interval of 5 K, a thermal conductivity of 50 W/(m·K), an isobaric heat capacity of 2000 J/(kg·K), a density of 1000 kg/m^3^, and a convective heat transfer coefficient of 10 W/(m^2^·K), and the other parameters remained unchanged while one particular parameter was studied.

### 4.1. The Effect of the Quantity of Phase Change Materials

In order to compare the effect of the number of PCMs, the influence of the height of the PCM heat sink on the temperature difference between the high and low-temperature ends, open circuit voltage, temperature difference per unit mass of PCM, output power, and output power density were investigated.

**Figure 3:**
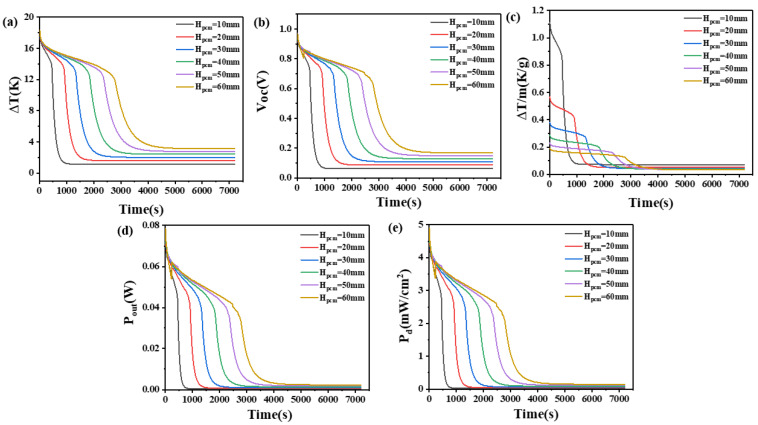
The temperature difference (**a**), open circuit voltage (**b**), temperature difference per unit mass of PCM (**c**), output power (**d**), and output power density (**e**) of the device versus time and height of PCM heat sink when the thermoelectric leg height is 1.6 mm.

**Figure 4:**
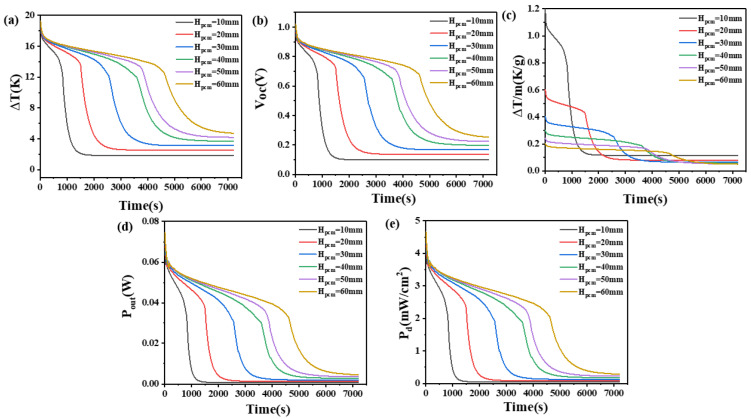
The temperature difference (**a**), open circuit voltage (**b**), temperature difference per unit mass of PCM (**c**), output power (**d**), and output power density (**e**) of the device versus time and height of PCM heat sink when the thermoelectric leg height is 2.7 mm.

The results are shown in Figure 3 and Figure 4. It can be seen from Figure 3a,b that the temperature difference across the device increases with an increase in the quantity of PCM. When the height of the thermoelectric leg is 1.6 mm and the height of the PCM is only 10 mm, the device can maintain a small temperature difference for a short time. At 1000 s, it can only maintain a temperature difference of 1.2 K, an open circuit voltage of 0.067 V, an output power of 0.3 mW, and a power density of 23 μW/cm^2^. Afterward, it remains basically unchanged. However, as the height of the PCM reaches 60 mm, the device exhibits a significant improvement in its ability to maintain high-temperature differences. Prior to 4000 s, it can maintain a temperature difference ranging from 17 K to 3.6 K, corresponding to a voltage range of 0.96 V to 0.19 V, an output power range of 78 mW to 3.18 mW, and a power density range of 4.9 mW/cm^2^ to 0.2 mW/cm^2^. Moreover, it can maintain a temperature difference of approximately 3.2 K, a voltage of about 0.17 V, an output power of 2.4 mW, and a power density of 0.15 mW/cm^2^ for the subsequent time. This suggests that a larger quantity of PCM is desirable; however, excessive PCM can increase the weight of the device, thus decreasing portability. Hence, this paper considered the thermal dissipation capacity per unit mass of the PCM. From Figure 3c, it can be seen that when the height of the PCM is 10 mm and the height of the thermoelectric leg is 1.6 mm, the temperature difference per unit mass of PCM can be maintained at 1.1–0.6 K/g for the first 500 s. For a PCM heat sink with a height of 60 mm, In the first 500 s, the temperature difference per unit mass of PCM can only be maintained at 0.2–0.16 K/g.

When the height of the thermoelectric leg is set at 2.7 mm, it can be seen from Figure 4 that the temperature difference across the thermoelectric device, open circuit voltage, temperature difference maintained by the PCM per unit mass, output power, and power density exhibit similar variations to those observed at a thermoelectric leg height of 1.6 mm. However, differences between the two are also evident. Specifically, with an increase in the thermoelectric leg height under certain volume conditions of the PCM, the time for the device to maintain the maximum temperature difference is significantly prolonged. For example, when the height of the PCM is set at 60 mm, the temperature difference of 12–18 K is maintained and increases from 2760 s, observed with a thermoelectric leg length of 1.6 mm to 4680 s. The corresponding duration for maintaining the respective high voltage and unit mass temperature difference also experiences a significant increase, as shown in Figure 4a. Moreover, at a PCM height of 60 mm, it is possible to maintain temperature differences above 14 K, open circuit voltages exceeding 0.74 V, output powers exceeding 0.04 W, and power density exceeding 2.5 mW/cm^2^ for 3900 s. Even at 7200 s, temperature differences of 4.7 V or higher, open circuit voltages exceeding 0.25 V, output powers exceeding 4.57 mW, and power density exceeding 0.29 mW/cm^2^ can still be maintained. In Figure 4c, it can be seen that when the height of the PCM is set at 10 mm, a unit mass of the PCM can maintain a temperature difference of 1.1 K–0.5 K/g for 930 s when the thermoelectric leg height is 2.7 mm. This is obviously superior to the heat dissipation performance per unit mass under other conditions. When the height of the PCM is set at 60 mm, a unit mass of the PCM can maintain a temperature difference of approximately 0.13 K/g for nearly 4500 s. This corresponds to a device open circuit voltage of 0.69 V, an output power of 34.6 mW, and a power density of 2.16 mW/cm^2^, which cannot be achieved by PCM under other heights.

Consequently, the size of the thermoelectric leg plays a very important role in maintaining a large temperature difference for a long enough time and the corresponding large open circuit voltage. Considering the long-term heat dissipation efficiency and wearing comfort, a thermoelectric leg size of 2.7 mm and a PCM height of H_pcm_ = 40 mm are considered appropriate in practical applications.

### 4.2. The Effect of Density of Phase Change Materials

Due to PCM with a density below 600 kg/m^3^ being rare and difficult to prepare, and the inconvenience associated with high density, we investigated the impact of PCM density, ranging from 600 to 1400 kg/m^3^, on the temperature difference between the high and low ends of the device, open circuit voltage, temperature difference maintained per unit mass of PCM, output power, and output power density, while keeping other parameters of the PCM constant.

**Figure 5:**
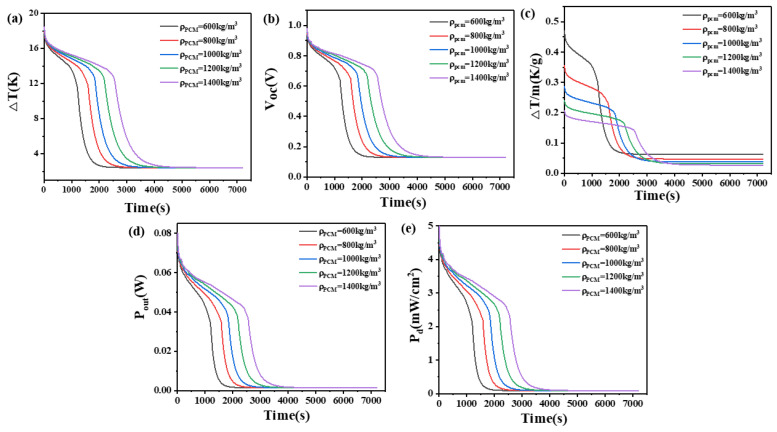
The temperature difference (**a**), open circuit voltage (**b**), temperature difference per unit mass of PCM (**c**), output power (**d**), and output power density (**e**) of the device versus time and density of PCM when the thermoelectric leg height is 1.6 mm.

**Figure 6:**
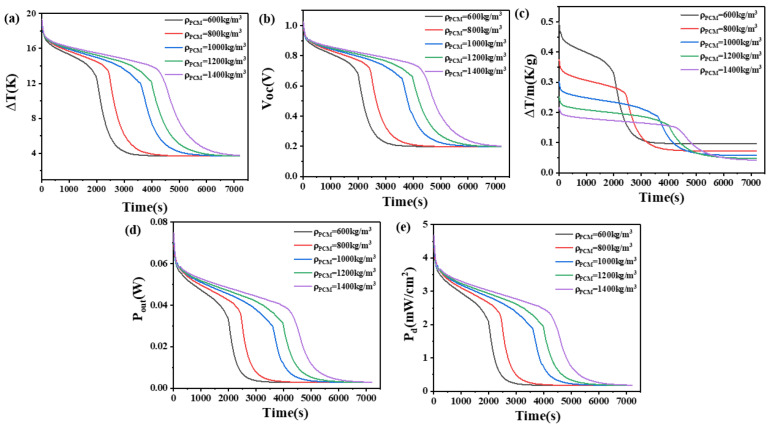
The temperature difference (**a**), open circuit voltage (**b**), temperature difference per unit mass of PCM (**c**), output power (**d**), and output power density (**e**) of the device versus time and density of PCM when the thermoelectric leg height is 2.7 mm.

The results are presented in Figure 5 and Figure 6. From Figure 5, it can be seen that when the height of the thermoelectric leg is 1.6 mm and the density of the PCM is 600 kg/m^3^, a temperature difference of 17–12 K can be maintained for 1170 s. Meanwhile, the open circuit voltage ranges from 0.98 V to 0.8 V, the output power ranges from 76.2 mW to 35.8 mW, and the output power density ranges from 4.76 mW/cm^2^ to 2.24 mW/cm^2^. At 2000 s, the temperature difference decreases to 2.6 K, corresponding to an open circuit voltage of 0.14 V, an output power of 1.7 mW, and an output power density of 0.11 mW/cm^2^. When the density increases to 1400 kg/m^3^, a temperature difference of 17–12 K can be maintained for 2570 s, reaching a temperature difference of 2.7 K at 4000 s. The corresponding open circuit voltage is approximately 0.15 V. When the height of the thermoelectric leg is 2.7 mm, a density of 600 kg/m^3^ allows for a temperature difference of 17–12 K to be maintained for 2030 s, with an open circuit voltage ranging from 0.98 V to 0.8 V. With an increased density of 1400 kg/m^3^, the temperature difference of 17–12 K can be maintained for 4480 s, reaching a temperature difference of 4 K at 6500 s. Meanwhile, the open circuit voltage is 0.21 V, the output power is 3.28 mW, and the output power density is 0.2 mW/cm^2^.

Figure 6 demonstrates that a higher density of PCM allows for a larger temperature difference to be maintained. This is because, with a constant volume, higher density correlates to a greater mass of PCM, resulting in a larger latent heat and more heat absorption capacity. However, in practical applications, the increase in density will increase the user’s burden and reduce the wearing comfort. Thus, we compared the ability of PCMs to maintain temperature differences per unit mass at different densities. It can be seen from Figure 6 that PCMs with different unit masses have their own advantages at different times. When the thermoelectric leg height is 1.6 mm, the PCM with a density of 600 kg/m^3^ can maintain a temperature difference of 0.45 K/g initially and experiences a performance decline around 1240 s. The PCM with a density of 800 kg/m^3^ has the best performance in a period of time after this stage. The same rule applies to devices with a thermoelectric leg height of 2.7 mm: for different densities of PCM, those with higher density exhibit improved performance in later time stages. Considering all time periods, PCM with a density of 600 kg/m^3^ maintains the largest temperature difference, achieving the highest performance for both the initial phase and the later time stages, which is a suitable choice.

### 4.3. The Effect of Phase Transition Temperature on Phase Change Materials

To investigate the impact of different phase transition temperatures on the performance of PCM heat sinks, the initial temperature of the PCM was set to 289.15 K. The effects on the temperature difference, open circuit voltage, output power, and power density at the two ends of the thermoelectric legs with heights of 1.6 mm and 2.7 mm were studied.

**Figure 7:**
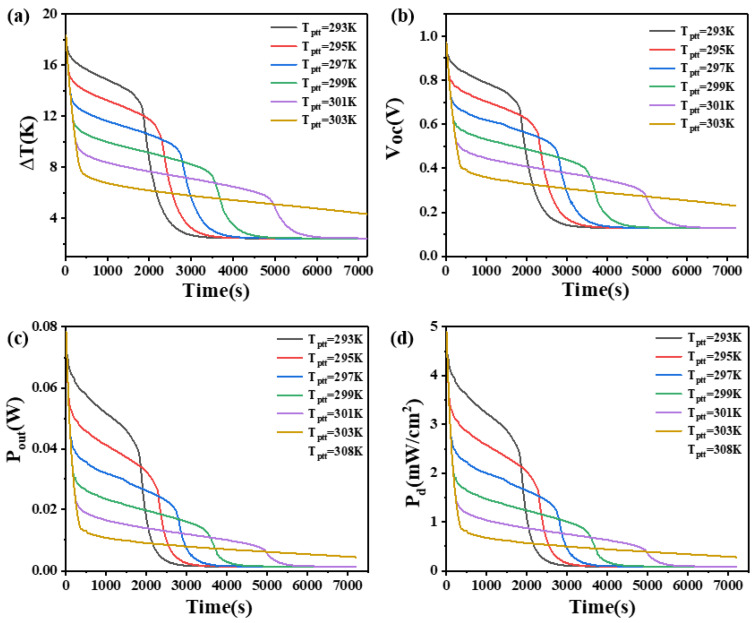
The temperature difference (**a**), open circuit voltage (**b**), output power (**c**), and output power density (**d**) of the device versus time and phase transition temperature of PCM when the thermoelectric leg height is 1.6 mm.

**Figure 8:**
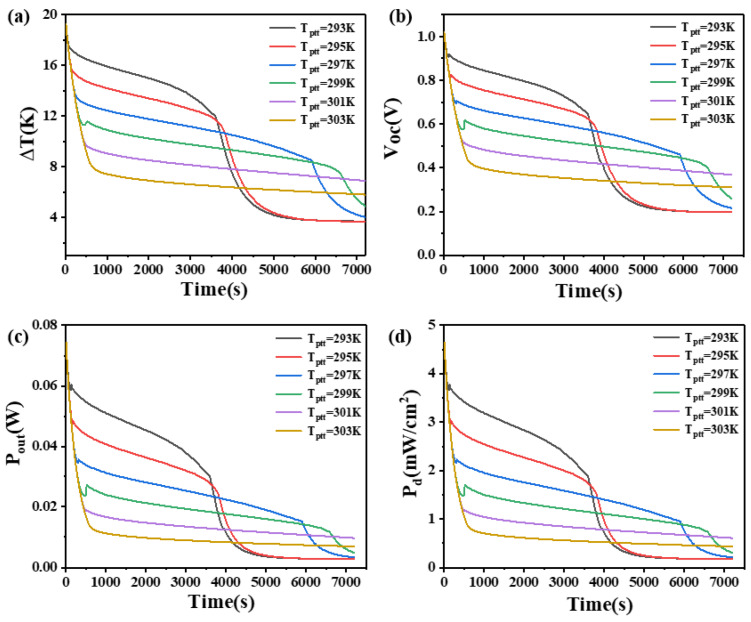
The temperature difference (**a**), open circuit voltage (**b**), output power (**c**), and output power density (**d**) of the device versus time and phase transition temperature of PCM when the thermoelectric leg height is 2.7 mm.

The results are shown in Figure 7 and Figure 8. From Figure 7, it can be seen that when the thermoelectric leg height is 1.6 mm, the PCM with a lower phase transition temperature initially functions as a heat sink, resulting in better heat dissipation within a shorter duration. It can maintain a larger temperature difference, with the PCM at 293 K maintaining a temperature difference of 17 K–13 K in the first 30 min, an open circuit voltage of 0.96 V–0.68 V, an output power of 78.3 mW–39.3 mW, and an output power density of 4.89 mW/cm^2^–2.46 mW/cm^2^. At 3000 s, the output power of the device is basically constant, corresponding to a temperature difference of 2.7 K, an open circuit voltage of 0.14 V, an output power of 1.7 mW, and an output power density of 0.11 mW/cm^2^. However, as the phase transition temperature of the PCM increases, the temperature point at which the PCM completely melts also increases. In Figure 7, the time point when the temperature difference begins to decline rapidly is increasing constantly. The phase transition temperature determines the maximum temperature difference that the PCM can maintain. The lower the phase transition temperature, the larger the temperature difference that can be maintained, but for a shorter duration. The PCM with a higher phase transition temperature can maintain a relatively moderate temperature difference for a long time. The high-temperature difference maintained by the PCM is inversely proportional to the time to maintain the high-temperature difference. When the phase transition temperature is 303 K, the PCM does not completely melt within two hours. Although it can only maintain a temperature difference of 7.6 K and an open circuit voltage of 0.4 V at 400 s, it can continuously maintain a temperature difference of at least 4.3 K in the following two hours, corresponding to an open circuit voltage of 0.23 V, an output power of 4.58 mW, and an output power density of 0.29 mW/cm^2^. If short-term device operation is desired, PCMs with a lower phase transition temperature of 293 K are more suitable. If higher power output is required within 2 h, PCMs with a phase transition temperature of 303 K are more appropriate. Besides, it is appropriate to select more than 2 kinds of PCMs to optimize the relationship. When the thermoelectric leg height is 2.7 mm, the rule of the phase transition temperature remains similar when the height of the thermoelectric leg is 1.6 mm, but there are some differences. Firstly, the rate of temperature difference decline at the ends of the thermoelectric device significantly decreases. Secondly, the duration of maintaining a large temperature difference obviously increases. For example, when the phase transition temperature of the PCM is 301 K and 303 K, the initial temperature difference decreases rapidly at approximately 440 s and 640 s, respectively, reducing to 10 K and 8 K. It can maintain temperature differences of 10 K–8 K and 8 K–6 K within 7200 s, where the PCM does not melt completely. The reason is that the higher thermoelectric leg height results in a lower temperature at the cold end so that it is less than the temperature required for the complete melting of the PCM. Correspondingly, the open circuit voltage ranges between 0.5 V–0.4 V and 0.4 V–0.3 V. When the phase transition temperature of the PCM is 297 K, the temperature difference decreases from 18.5 K to 13.5 K at 300 s, reaching 10 K at 4600 s. The corresponding open circuit voltage is 0.53 V, and after 6000 s, the temperature difference decreases to 9 K with an open circuit voltage of 0.5 V. If the device needs to operate within the first 4000 s, the PCM with a phase transition temperature of 293 K is most suitable. If continuous operation within 7200 s is required, the PCM with a phase transition temperature of 297 K is more appropriate. These findings indicate that different thermoelectric leg heights have varying requirements for the phase transition temperature of the PCM, and an increase in thermoelectric leg height benefits the enhancement of the temperature difference and open circuit voltage at the ends of the thermoelectric device.

### 4.4. The Effect of Thermal Conductivity of Phase Change Materials

**Figure 9:**
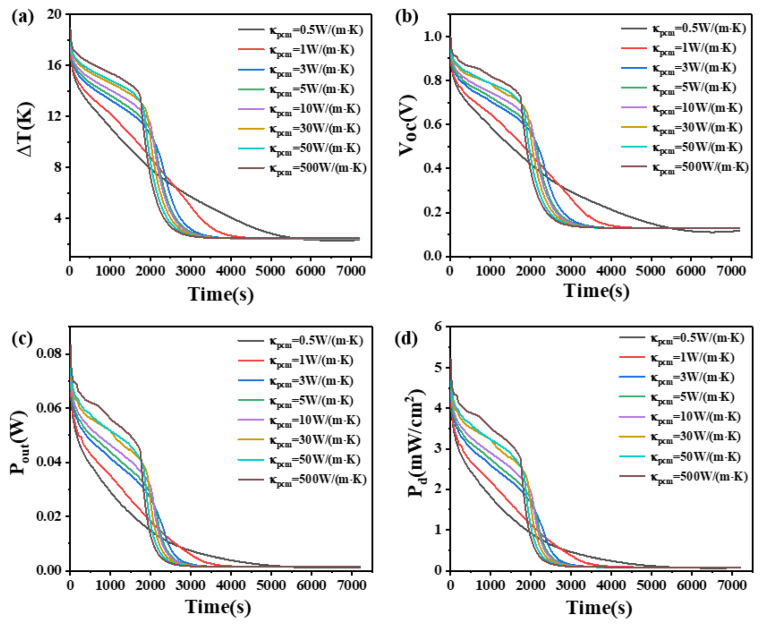
The temperature difference (**a**), open circuit voltage (**b**), output power (**c**), and output power density (**d**) of the device versus time and thermal conductivity of PCM when the thermoelectric leg height is 1.6 mm.

**Figure 10:**
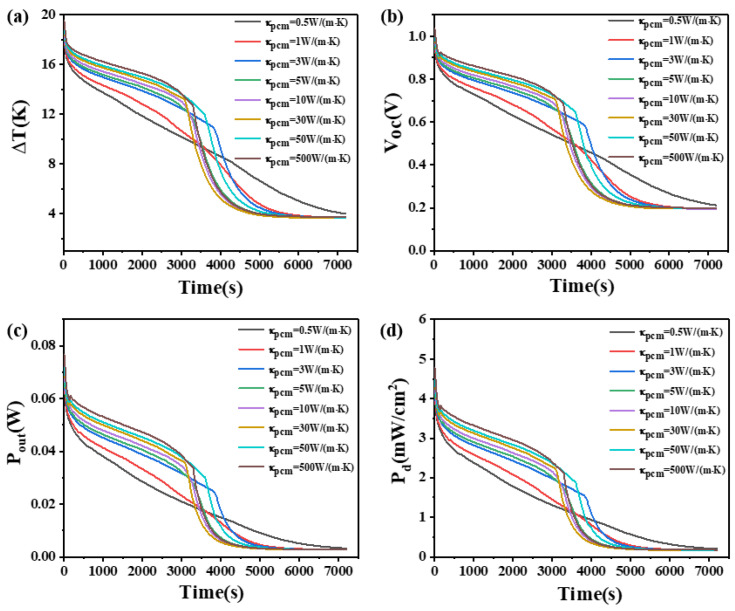
The temperature difference (**a**), open circuit voltage (**b**), output power (**c**), and output power density (**d**) of the device versus time and thermal conductivity of PCM when the thermoelectric leg height is 2.7 mm.

This study investigates the influence of the thermal conductivity of PCMs on the temperature difference, open circuit voltage, output power, and power density. The results are shown in Figure 9 and Figure 10. Figure 9 demonstrates that when the height of the thermoelectric leg is 1.6 mm and the thermal conductivity of the PCM is 0.5 W/(m·K), the temperature difference of the device decreases and the rate of decline has remained unchanged. The effect of PCM is relatively small. However, when the thermal conductivity of the PCM is increased to 5 W/(m·K), the temperature difference that the device can maintain is greatly improved. The device maintains a temperature difference ranging from 17 K to 11 K for a duration of approximately 2000 s, corresponding to an open circuit voltage ranging from 0.94 V to 0.58 V, an output power ranging from 72.9 mW to 28.1 mW, and an output power density ranging from 4.56 mW/cm^2^ to 1.75 mW/cm^2^. Furthermore, with a thermal conductivity of 500 W/(m·K) for the PCM, the device can maintain a temperature difference between 18.8 K and 7.4 K for the first 2000 s, exhibiting an open circuit voltage ranging from 1.0 V to 0.4 V, an output power ranging from 83.3 mW to 12.9 mW, and an output power density ranging from 5.21 mW/cm^2^ to 0.8 mW/cm^2^. Figure 10 shows that when the height of the thermoelectric leg is 2.7 mm and the thermal conductivity of the PCM is 5 W/(m·K), a temperature difference of 17–11 K can be maintained for approximately 3380 s. This corresponds to an open circuit voltage ranging from 1.0 V to 0.59 V, an output power ranging from 71.9 mW to 25.1 mW, and an output power density ranging from 4.5 mW/cm^2^ to 1.57 mW/cm^2^. It is obvious that when the thermal conductivity of the PCM continues to increase, although the temperature difference of the device also increases, the increasing effect is reduced, so the thermal conductivity of phase change materials needs to be selected according to the time of use. Additionally, it can be seen that when the height of the thermoelectric leg is 1.6 mm, greater thermal conductivity of the PCM leads to a smaller temperature difference after 2500 s, but for a height of 2.7 mm, higher thermal conductivity decreases the temperature difference of the device after 4000 s. The thermal conductivity of the PCM generally ranges from 0.1 W/(m·K) to 0.6 W/(m·K). By appropriately doping high thermal conductivity materials such as carbon nanotubes, the composite thermal conductivity of the PCM can reach around several tens of W/(m·K). By comprehensively analyzing the effects of different thermal conductivities on the temperature difference and duration of maintenance for the device, as presented in Figure 9 and Figure 10, it is appropriate to select a PCM with a thermal conductivity of 50 W/(m·K) for practical applications.

The heights of thermoelectric leg devices are as follows:

### 4.5. The Effect of Phase Transition Temperature Interval of Phase Change Materials

The phase transition temperature interval is the difference between the two temperatures at which the PCM begins its phase transition from one temperature to another. This study investigates the impact of the phase transition temperature interval on the temperature difference, open circuit voltage, output power, and power density at both ends of two heights of thermoelectric devices.

**Figure 11:**
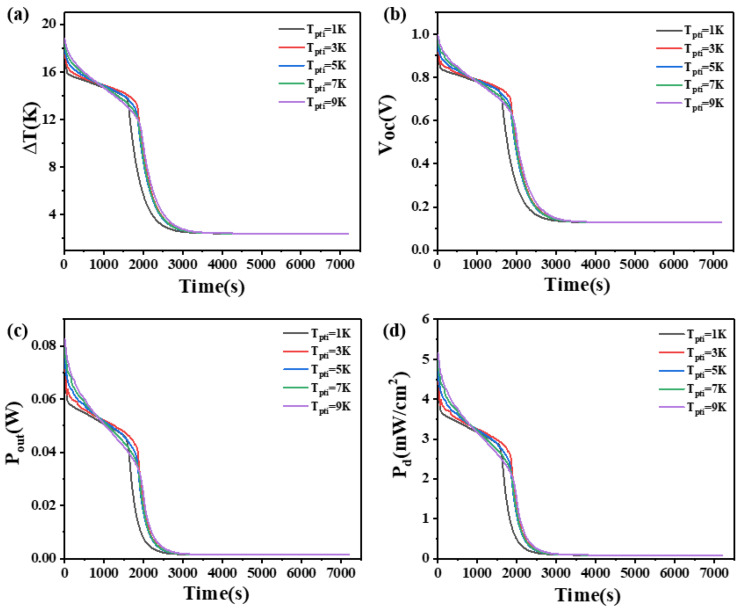
The temperature difference (**a**), open circuit voltage (**b**), output power (**c**), and output power density (**d**) of the device versus time and phase transition temperature interval of PCM when the thermoelectric leg height is 1.6 mm.

**Figure 12:**
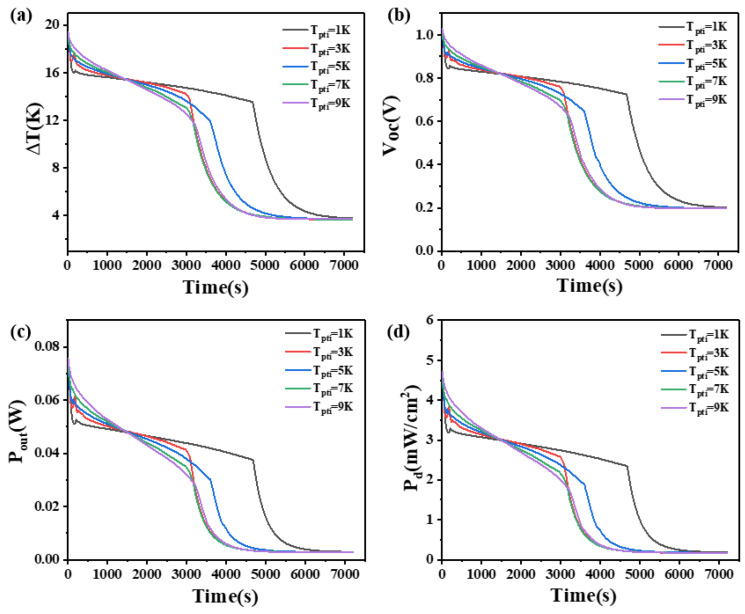
The temperature difference (**a**), open circuit voltage (**b**), output power (**c**), and output power density (**d**) of the device versus time and phase transition temperature interval of PCM when the thermoelectric leg height is 2.7 mm.

The results are presented in Figure 11 and Figure 12. From Figure 11, it is obvious that when the height of the thermoelectric leg is 1.6 mm, the device’s temperature difference increases with an increase in the phase transition temperature interval within 850 s. However, after 850 s, the temperature difference decreases rapidly as the phase transition temperature interval increases. The critical transition time is 850 s. Before 850 s, a larger phase transition temperature interval in the PCM leads to a greater maintained temperature difference. Inversely, after 850 s, a larger phase transition temperature interval results in a smaller maintained temperature difference. The reason is that when the latent heat of the PCM is constant, the heat absorbed by the PCM upon completion of the transition remains constant. Hence, a larger phase transition temperature interval leads to a longer duration during the PCM works. During the initial 2000 s, a PCM with a phase transition temperature interval of 1 K causes a rapid decrease in the device’s temperature difference from 17.7 K to 5.7 K, open circuit voltage from 0.93 V to 0.3 V, output power from 78.3 mW to 7.69 mW, and power density from 4.89 mW/cm^2^ to 0.48 mW/cm^2^. In contrast, a PCM with a phase transition temperature interval of 9 K maintains a temperature difference of at least 10 K, an open circuit voltage of at least 0.53 V, an output power of at least 23.7 mW, and a power density of at least 1.48 mW/cm^2^. For Figure 11, the point with the largest slope near the 2000 s represents the complete melting of the PCM, as it no longer works as a heat sink. Figure 11 illustrates that, with the increase in the phase transition temperature interval, the time point of the largest slope point is also later, which can prove that the phase transition temperature interval does play a role. When the height of the thermoelectric leg is 2.7 mm, the temperature difference of the device also conforms to this rule in the first 3000 s. During the first half of this period (1500 s), a larger phase transition temperature interval in the PCM corresponds to a greater maintained temperature difference, but the opposite is observed in the latter half. After 3000 s, it is obvious that the PCM with a phase transition temperature interval of 1 K exhibits better heat dissipation compared to other phase transition temperature intervals. It maintains a temperature difference of at least 13 K, an open circuit voltage of at least 0.7 V, an output power of at least 35.5 mW, and a power density of at least 2.22 mW/cm^2^ up to 4700 s.

Figure 11 and Figure 12 illustrate a one-to-one correspondence between the output voltage and the corresponding temperature difference, as the output voltage is proportional to the temperature difference. For the device with a thermoelectric leg height of 1.6 mm, the PCM with a phase transition temperature interval of 1 K exhibits the fastest melting speed, resulting in a relatively small maintained temperature difference and inferior performance, making it unsuitable for selection. After the PCM is completely melted. the PCM with a phase transition temperature interval of 9 K maintains the largest temperature difference and the highest average temperature difference throughout the entire time period, making it suitable for practical applications. On the other hand, when the height of the thermoelectric leg is 2.7 mm, the PCM with a phase transition temperature interval of 1 K should be chosen. This indicates that different heights of the thermoelectric leg have different requirements for the phase transition temperature interval of the PCM. Additionally, an increased height of the thermoelectric leg facilitates an improvement in the temperature difference and open circuit voltage at both ends of the thermoelectric device.

### 4.6. The Effect of Latent Heat on Phase Change Materials

**Figure 13:**
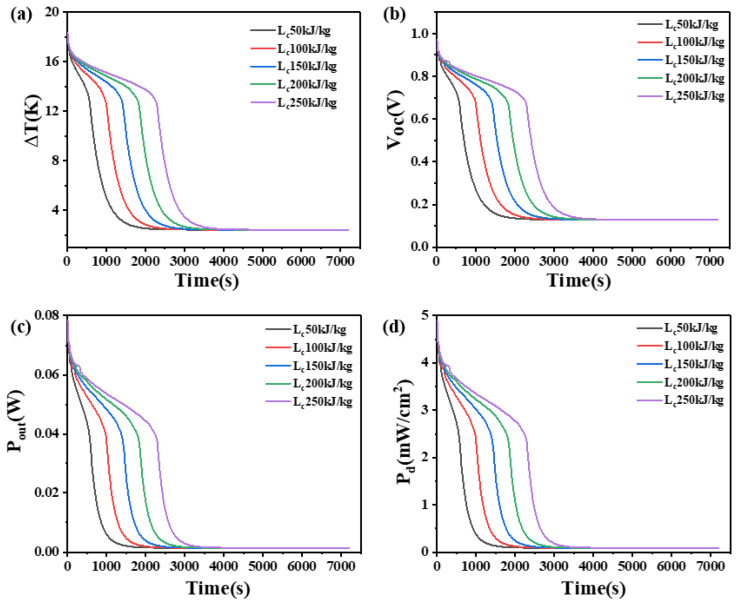
The temperature difference (**a**), open circuit voltage (**b**), output power (**c**), and output power density (**d**) of the device versus time and latent heat of PCM when the thermoelectric leg height is 1.6 mm.

**Figure 14:**
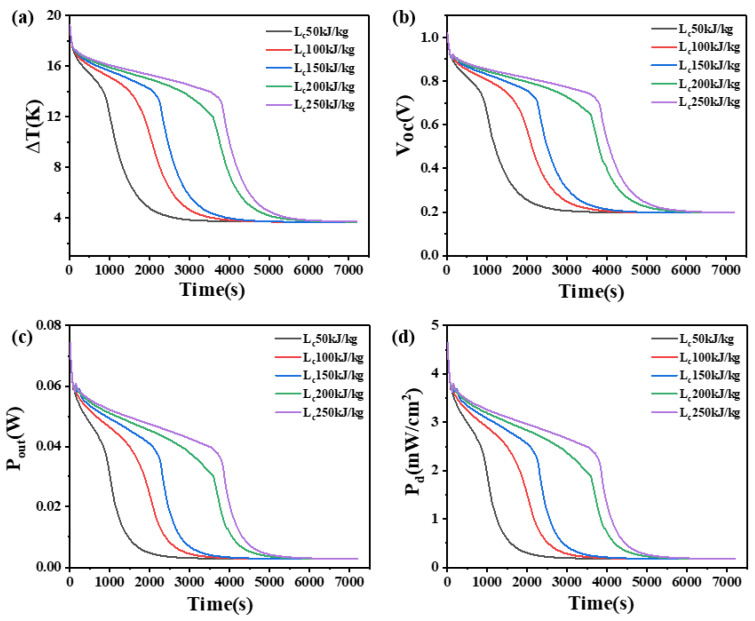
The temperature difference (**a**), open circuit voltage (**b**), output power (**c**), and output power density (**d**) of the device versus time and latent heat of PCM when the thermoelectric leg height is 2.7 mm.

The latent heat of PCM refers to the quantity of heat absorbed or released by a unit mass of material under isothermal and isobaric conditions from one phase to another phase. This paper investigates the influence of the latent heat of PCM on the temperature difference, open circuit voltage, output power, and power density at the two ends of two heights of thermoelectric devices, as shown in Figure 13 and Figure 14.

From Figure 13, it can be seen that when the height of the thermoelectric leg is 1.6 mm, a greater latent heat in the PCM allows for a higher and longer sustainable temperature difference. With a latent heat of 50 kJ/kg, the device can only maintain a temperature difference of 14 K, an open circuit voltage of 0.75 V, an output power of 46.4 mW, and an output power density of 2.9 mW/cm^2^ for 450 s. At 1000 s, the temperature difference can only be maintained at 5 K, open circuit voltage at 0.27 V, output power at 6.11 mW, and output power density at 0.38 mW/cm^2^. However, when the latent heat of PCM is 250 kJ/kg, a temperature difference of 14 K and an open circuit voltage of 0.75 V can be maintained for the 1800 s, indicating a significant improvement in performance compared to the PCM with the latent heat of 50 kJ/kg. When the PCM is completely melted, the trend of the overall temperature difference of the device and the final temperature difference are the same, which is 2.418 K and the open circuit voltage is 0.13 V, the output power is 1.4 mW, and the output power density is 0.087 mW/cm^2^. At this point, the material state is the same, the latent heat no longer plays a role.

As shown in Figure 14, when the height of the thermoelectric leg is 2.7 mm, an obvious reduction in the rate of temperature difference decreases and a significant extension in the duration of sustaining a large temperature difference can be observed compared to the 1.6 mm leg height. For instance, with a latent heat of 250 kJ/kg, it takes 3720 s to lower the temperature difference from 18.5 K to 13.5 K, while it only takes 2110 s for the thermoelectric leg height of 1.6 mm. Similarly, the corresponding open circuit voltage decreases from 1.0 V to 0.73 V, indicating the ability to maintain an open circuit voltage of at least 0.73 V for 3720 s, an output power of at least 37.7 mW, and a power density of at least 2.36 mW/cm^2^. Furthermore, it is obvious that the temperature difference at both ends of the device, the duration of maintaining a large temperature difference, the open circuit voltage, the output power, and the power density all increase with the increase in latent heat of PCM. For example, when the latent heat of PCM increases from 150 kJ/kg to 200 kJ/kg, the temperature difference across the generator device significantly improves, with a temperature difference of at least 12 K, an open circuit voltage of at least 0.65 V, an output power of at least 30.2 mW, and an output power density of at least 1.89 mW/cm^2^ being maintained for 3600 s. In practical applications, it is suitable to choose PCMs with higher latent heat.

### 4.7. The Effect of Isobaric Heat Capacity on Phase Change Materials

**Figure 15:**
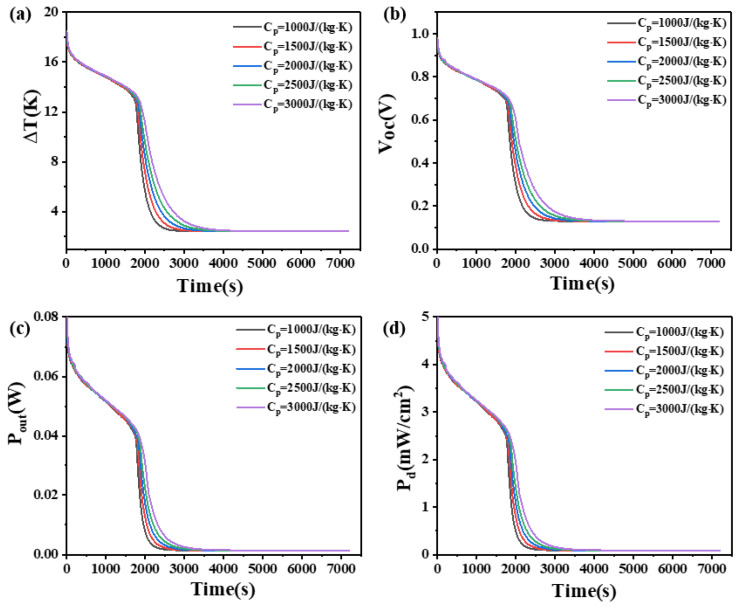
The temperature difference (**a**), open circuit voltage (**b**), output power (**c**), and output power density (**d**) of the device versus time and isobaric heat capacity of PCM when the thermoelectric leg height is 1.6 mm.

**Figure 16:**
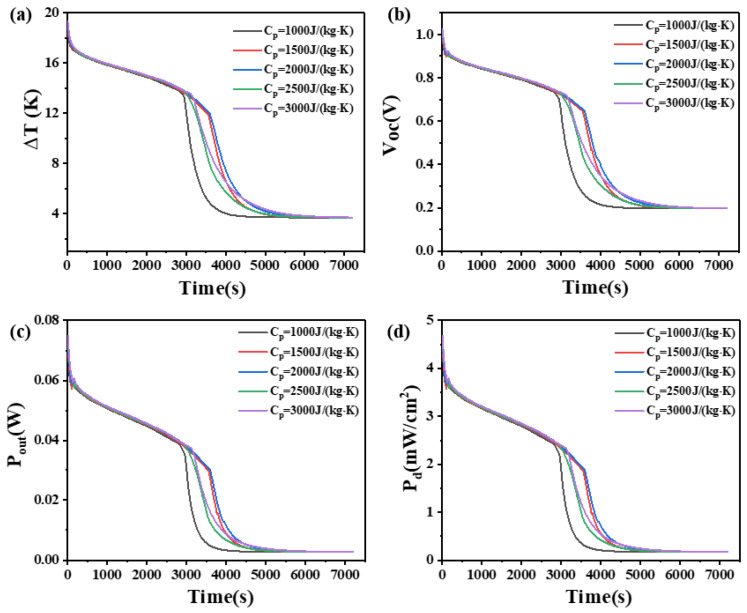
The temperature difference (**a**), open circuit voltage (**b**), output power (**c**), and output power density (**d**) of the device versus time and isobaric heat capacity of PCM when the thermoelectric leg height is 2.7 mm.

The isobaric heat capacity refers to the quantity of heat absorbed per unit mass of a material when its temperature increases by 1 K, while the pressure remains constant. This study investigates the influence of PCMs with different isobaric heat capacities (1000, 1500, 2000, 2500, and 3000 J/(kg·K)) on the temperature difference, open circuit voltage, output power, and power density of two heights of thermoelectric devices. The results are shown in Figure 15 and Figure 16.

As shown in Figure 15, for a leg height of 1.6 mm, within 2500 s, the temperature difference increases with an increase in the isobaric heat capacity. After 2500 s, the temperature difference of the device decreases rapidly, and the rate of decrease slows down with increasing isobaric heat capacity. After the 3500 s, the temperature differences for different heat capacities become almost constant, with no significant differences. At 2500 s, the temperature differences that they can maintain are 2.675 K, 3.093 K, 3.646 K, 4.299 K, and 5.133 K, respectively. The corresponding open circuit voltages are 0.14 V, 0.16 V, 0.19 V, 0.23 V, and 0.27 V, respectively. The corresponding output powers are 1.71 mW, 2.28 mW, 3.17 mW, 4.4 mW, and 6.3 mW, respectively. The corresponding power density are 0.11 mW/cm^2^, 0.14 mW/cm^2^, 0.19 mW/cm^2^, 0.26 mW/cm^2^, and 0.62 mW/cm^2^. It can be seen that the differences are not significant at this stage.

On the other hand, for a leg height of 2.7 mm, as shown in Figure 16, the variations in temperature difference and other parameters before 3000 s and after 6000 s are similar to those of the 1.6 mm-high device before 2000 s and after 3500 s, respectively. However, between 3000 s and 6000 s, the rules are different for the two leg heights. Specifically, between 3500 s and 4000 s, the rate of decrease in temperature difference with increasing heat capacity initially decreases, reaches a minimum at Cp = 2000 J/(kg·K), and then increases. The device with PCM has an isobaric heat capacity of 2000 J/(kg·K) and achieves the maximum temperature difference at 4000 s, which is 7.5 K, with an open circuit voltage of 0.4 V, an output power of 11.6 mW, and a power density of 0.72 mW/cm^2^.

The reason can be seen in Figure 15. When the leg height is 1.6 mm, an increase in the isobaric heat capacity leads to a larger temperature difference that the PCM can maintain for a longer period. From the initial stage to the complete melting of the PCM, the elevated temperatures of the PCMs are the same, and the PCM with high isobaric heat capacity and the PCM with low isobaric heat capacity play the same role. After complete melting, the PCM with high isobaric heat capacity absorbs more heat, but it is not obvious. Compared with the changes in other parameters, the change in isobaric heat capacity has little effect on the heat dissipation capacity of PCMs.

Figure 16 illustrates that, for a leg height of 2.7 mm, the rules of temperature difference and open circuit voltage of the thermoelectric device are different from those of the 1.6 mm-high device. The device with PCM that isobaric heat capacity is 2000 J/(kg·K) exhibits the maximum temperature difference, and further increasing the isobaric heat capacity of the PCM results in poor heat dissipation. As the phase change material absorbs more heat, its temperature begins to approach its phase change temperature. When a phase change occurs, although the phase change material continues to absorb heat, this heat is used for the phase change process, rather than increasing the temperature. As a result, the temperature at the cold end of the thermoelectric generator may no longer drop, resulting in a reduction in temperature difference. This indicates that different leg heights have different requirements for the isobaric heat capacity of the PCM and increasing the leg height is beneficial for improving the temperature difference and open circuit voltage of the device.

### 4.8. The Effect of Convective Heat Transfer Coefficient

**Figure 17:**
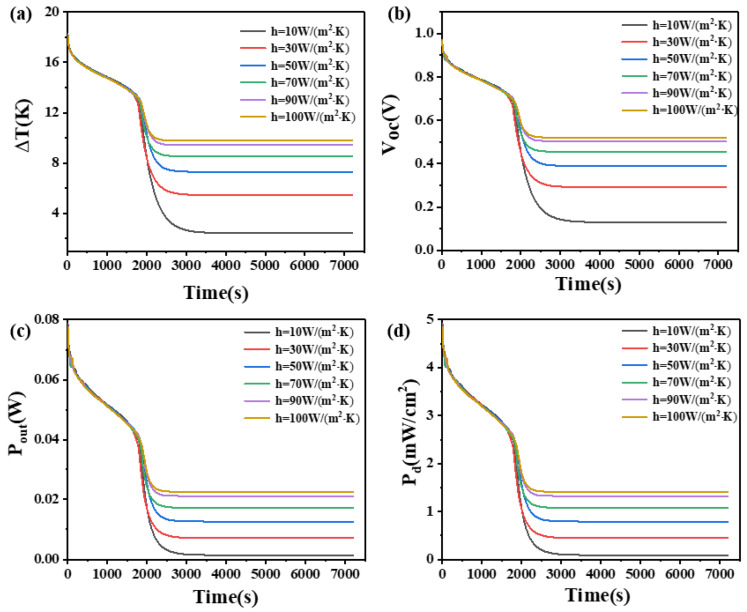
The temperature difference (**a**), open circuit voltage (**b**), output power (**c**), and output power density (**d**) of the device versus time and convective heat transfer coefficient when the thermoelectric leg height is 1.6 mm.

**Figure 18:**
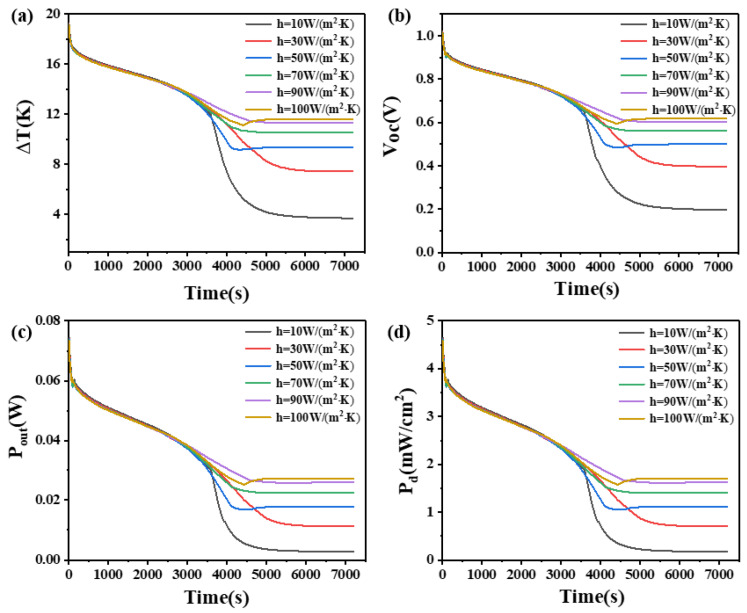
The temperature difference (**a**), open circuit voltage (**b**), output power (**c**), and output power density (**d**) of the device versus time and convective heat transfer coefficient when the thermoelectric leg height is 2.7 mm.

Due to people in reality are not completely stationary, the heat dissipation between devices and their environment is not only governed by natural convection. The natural convection heat transfer coefficient typically ranges between 5 W/(m^2^·K) and 25 W/(m^2^·K) [25,26]. However, during activities such as walking or running, the forced convection heat transfer coefficient of gas can reach 20 W/(m^2^·K)–300 W/(m^2^·K). This study investigates the effects of different convective heat transfer coefficients (10, 30, 50, 70, 90, and 100 W/(m^2^·K)) on the temperature difference, open circuit voltage, output power, and power density at both ends of two thermoelectric leg devices with different heights. The results are presented in Figure 17 and Figure 18.

From Figure 17, it can be seen that when the thermoelectric leg height is 1.6 mm, the temperature difference during device operation increases with an increase in the convective heat transfer coefficient. Different convective heat transfer coefficients exhibit similar influences on device performance during the initial 2000 s. However, significant differences become apparent once the PCM is fully melted (in the 2000 s). As the convective heat transfer coefficient increases, the rate of temperature difference reduction decreases, leading to an extended duration of large temperature differences. For instance, when the convective heat transfer coefficient is 10 W/(m^2^·K), the device can only maintain a temperature difference of 2.7 K, an open circuit voltage of 0.14 V, an output power of 1.7 mW, and a power density of 0.11 mW/cm^2^ after 3000 s. However, when the convective coefficient reaches 100 W/(m^2^·K), the device can maintain a temperature difference of at least 9.8 K, an open circuit voltage of at least 0.52 V, an output power of at least 22.6 mW, and a power density of at least 1.41 mW/cm^2^ within 7200 s. This highlights the substantial influence of convective heat transfer coefficients on the temperature difference and output performance of the device.

Figure 18 reveals that when the thermoelectric leg height is 2.7 mm, different convective heat transfer coefficients have a similar time dependence on the temperature difference and other parameters of the device at a height of 1.6 mm before 3500 s and after 4900 s, which means the effect on the performance of the device is the same, and the temperature difference of the device increases with the increase of the convective heat transfer coefficient, but the effect is not significant. However, between 3500 s and 4900 s or after the complete melting of the PCM, the convective heat transfer coefficient significantly affects the changes in temperature difference and exhibits different rules compared to the 1.6 mm device. With the increase of the convective heat transfer coefficient, the decrease rate of temperature difference decreases first, increases when the convective heat transfer coefficient is 50 W/(m^2^·K), and then decreases. When the convective heat transfer coefficient is 90 W/(m^2^·K), the decrease rate is the lowest, but after 4900 s, the decrease rate of temperature difference decreases with the increase of the convective heat transfer coefficient, so that the temperature difference is the largest when the convective heat transfer coefficient is 100 W/(m^2^·K). Overall, the device with a thermoelectric leg height of 2.7 mm exhibits a greater temperature difference and a longer duration of maintaining a large temperature difference compared to the 1.6 mm device. Similar to the 1.6 mm device, obvious differences become apparent after the complete melting of the PCM. For example, when the convective heat transfer coefficient is 10 W/(m^2^·K), the device can still maintain a temperature difference of 4 K, an open circuit voltage of 0.21 V, an output power of 3.3 mW, and a power density of 0.21 mW/cm^2^ after 5000 s, which is 1.6 times that observed in the 1.6 mm thermoelectric leg device. Comparing Figure 17 and Figure 18, the trend of the influence of different convective heat transfer coefficients on the temperature difference, open circuit voltage, output power, and power density at both ends of the two thermoelectric leg devices is roughly similar. Once the convective heat transfer coefficient reaches 50 W/(m^2^·K), further increases in the convective heat transfer coefficient have a limited effect on enhancing the temperature difference maintained by the two thermoelectric leg devices of different heights. In conclusion, it is obvious that improving the convective heat transfer coefficient is beneficial for maintaining the temperature difference in the device. Therefore, in practical applications, it is suitable to operate the device in an environment with the highest possible convective heat transfer coefficient.

## 5. Conclusions

This paper provides a concise overview of the current research status of PCM heat sinks in wearable thermoelectric generators. PCM heat sinks have the advantages of simplicity in structure, low cost, compactness, high latent heat capacity, and strong thermal energy storage capability. In this paper, COMSOL Multiphysics software was employed to simulate and calculate the output performance of wearable thermoelectric generators utilizing PCM as a heat sink. The key findings are as follows:

(1) The more the quantity of phase change materials, the greater the density, the greater the latent heat, the greater the height of the thermoelectric leg, and the greater the convective heat transfer coefficient, the better the performance of the heat sink. The height of the thermoelectric leg should be given priority in the selection.
(2) The phase change temperature of the PCMs determines the maximum temperature difference that can be maintained. PCMs with lower phase transition temperatures can maintain higher temperature differences in a short period, while those with higher phase transition temperatures can maintain a relatively stable temperature difference over a longer period. However, different heights of the thermoelectric leg require different phase transition temperatures. Alternatively, employing multiple PCMs can optimize their combined performance.
(3) The influence of thermal conductivity on PCMs resembles that of the phase transition temperature.
(4) When the height of the thermoelectric leg is 1.6 mm, the greater the isobaric heat capacity of the phase change material used, the better the output performance of the device. When the height of the thermoelectric leg is 2.7 mm, the isobaric heat capacity of the phase change material used is 2000 J/(kg·K), the better the output performance of the device.
(5) When the thermoelectric leg height is 1.6 mm, a PCM with a phase transition temperature interval of 9 K maintains the largest temperature difference, along with the greatest average temperature difference over the entire duration. However, when the thermoelectric leg height is 2.7 mm, a PCM with a phase transition temperature interval of 1 K should be selected because it can maintain a temperature difference of at least 13 K for the first 4700 s.

## Figures and Tables

**Table 1:** The size of the material.

Material	Length [mm]	Width [mm]	Height [mm]
Alumina	40	40	0.9
Thermoelectric legs	1.4	1.4	1.6/2.7
Copper guide strip	1.4	3.8	0.4
Wall of phase change material container	42	42	1
Phase change material	40	40	40

**Table 2:** The performance parameters of the material used in thermoelectric devices.

	P-Type Material	N-Type Material	Copper	Alumina	Phase Change Material	Thermoelectric Module
Isobaric heat Capacity (J/kg K)	154	154	385	900	2000	-
Density (kg/m^3^)	7700	7700	8960	2700	1000	-
Electrical conductivity (S/m)	σ (T)	σ (T)	5.998 × 10^7^	0	-	1/3(H_TL_ = 1.6 mm) 2/7(H_TL_ = 2.7 mm)
Seebeck coefficient (V/K)	α(T)	−α(T)	-	-	-	-
Thermal conductivity, (W/m^2^ K)	k(T)	k(T)	400	2	50	-
Relative permittivity	1	1	1	1	-	-

**Table 3:** Comparison table of calculation results of different grids.

	Number of Elements	Average Cell Quality	V_oc_ (t = 7200 s; V)	∆T (t = 7200 s; V)
extra fine	644,050	0.6647	0.12953	2.414
finer	331,144	0.651	0.12952	2.418
fine	179,556	0.6462	0.12949	2.422
normal	127,270	0.6321	0.12948	2.422
coarse	79,767	0.5843	0.12948	2.425
coarser	35,391	0.4929	0.12945	2.429

## Data Availability

The raw data supporting the conclusions of this article will be made available by the authors on request.

## Nomenclature

A	boundary area, m^2^
α_ab_	the Seebeck coefficient difference between the two materials, V/K
C_p_	heat capacity at constant pressure, J/(kg·K)
h	convective heat transfer coefficient, W/(m^2^·K)
H	height, m
k_c_	thermal conductivity of composite phase change materials, W/(m·K)
k_g_	thermal conductivity of graphene, W/(m·K)
L	length, m
L_c_	latent heat of composite phase change material, J/kg
L_pcm_	latent heat of phase change material, J/kg
$L_{1\to 2}$	latent heat, J/kg
n	unit vector
P_b_	heat rate, W
P_d_	theoretical output power density, W/m^2^
P_out_	theoretical output power, W
Q	heat source, W/m^3^
q	heat flux density, W/m^2^
Q_b_	heat rate per unit area, W
Qc	heat of the cold end of the thermoelectric generator, W
Q_h_	heat of the hot end of the thermoelectric generator, W
q_0_	the heat flux density in a certain direction, W/m^2^
Re	the internal resistance of thermoelectric generator, Ω
R_L_	load resistance, Ω
t	time, s
T	temperature, K
T_c_	cold end temperature, K
T_h_	hot end temperature, K
V	voltage, V
V_g_	the volume fraction of graphene
w	the mass fraction of graphene
W	width, m
ΔT	temperature difference, K
ΔV	seebeck voltage, V/K
*Greek symbols*	
α	electrical conductivity, S/m
η	efficiency
κ	thermal conductivity, W/m/K
ρ	density, kg/m^3^
μ	velocity field, m/s
θ	content of a certain phase
*Abbreviations*	
Bi_2_Te_3_	bismuth telluride
EG	expanded graphite
PCM	phase change material
TEG	thermoelectric generator
TED	thermoelectric device
TL	thermoelectric leg
*Subscripts*	
ext	exterior
oc	open circuit
pti	phase transition temperature interval
ptt	phase transition temperature
ted	thermoelastic damping
